# Suitability of Artificial Membranes in Lipolysis-Permeation Assays of Oral Lipid-Based Formulations

**DOI:** 10.1007/s11095-020-02833-9

**Published:** 2020-05-20

**Authors:** Oliver J. Hedge, Christel A. S. Bergström

**Affiliations:** 1 Department of Pharmacy, Uppsala University, Husargatan 3, Box 580, SE-75123 Uppsala, Sweden; 2 The Swedish Drug Delivery Center, Department of Pharmacy, Uppsala University, Uppsala, Sweden

**Keywords:** artificial membrane, digestion, lipid-based formulation, permeation, self-emulsifying oral drug-delivery systems

## Abstract

**Purpose:**

To evaluate the performance of artificial membranes in *in vitro* lipolysis-permeation assays useful for absorption studies of drugs loaded in lipid-based formulations (LBFs).

**Methods:**

Polycarbonate as well as PVDF filters were treated with hexadecane, or lecithin in *n*-dodecane solution (LiDo) to form artificial membranes. They were thereafter used as absorption membranes separating two compartments mimicking the luminal and serosal side of the intestine *in vitro*. Membranes were subjected to dispersions of an LBF that had been digested by porcine pancreatin and spiked with the membrane integrity marker Lucifer Yellow (LY). Three fenofibrate-loaded LBFs were used to explore the *in vivo* relevance of the assay.

**Results:**

Of the explored artificial membranes, only LiDo applied to PVDF was compatible with lipolysis by porcine pancreatin. Formulation ranking based on mass transfer in the LiDo model exposed was the same as drug release in single-compartment lipolysis. Ranking based on observed apparent permeability coefficients of fenofibrate with different LBFs were the same as those obtained in a cell-based model.

**Conclusions:**

The LiDo membrane was able to withstand lipolysis for a sufficient assay period. However, the assay with porcine pancreatin as digestive agent did not predict the *in vivo* ranking of the assayed formulations better than existing methods. Comparison with a Caco-2 based assay method nonetheless indicates that the *in vitro in vivo* relationship of this cell-free model could be improved with alternative digestive agents.

**Electronic supplementary material:**

The online version of this article (10.1007/s11095-020-02833-9) contains supplementary material, which is available to authorized users.

## Introduction

Artificial membranes can be a valuable tool for evaluating properties of drug compounds pertaining to barrier interactions (1). When properly used, they can give information about functional characteristics with increased cost-effectiveness and fewer ethical concerns than animal- or cell-based experiments. However, it is important to identify the limits of such membranes so that they can be used to produce valid experimental data. In the digestion process (lipolysis) of lipid-based formulations (LBFs), membranes will be subjected to a matrix consisting of surfactants and lipids, along with lipid digesting enzymes, which may affect the membrane structure and its integrity over time. In this study, the suitability of artificial membranes to enhance the *in vitro* lipolysis assay with simultaneous absorption (lipolysis-permeation assay) was investigated.

Between 2015 and 2019, the number of highly lipophilic (logP >5) New Molecular Entities (NMEs) accounted for approximately 15% of all NMEs intended for oral administration (Table S1, Supplementary Material). Moderately to highly lipophilic NMEs (logP >3) from 2015 to 2019 accounted for 49% (2). Highly lipophilic drug compounds often suffer from inter- and intraindividually variable bioavailability. Apart from metabolism, this variability can be caused largely by solubility issues and influenced by factors like gastrointestinal physiology, prandial state during the time of administration, and composition of the latest meal. Thus, it is important to consider the formulation of these APIs in such a way that their negative characteristics can be minimized.

LBFs such as self-emulsifying drug delivery systems (SEDDS) is a tool in the formulator’s toolbox for improving bioavailability for poorly water-soluble drugs, an alternative to other options such as salt formation, solid dispersion, amorphization, or complexation. By 2016, there were at least 35 LBF drug products approved by the FDA, but the rate of new LBFs coming out on the market has slowed down in recent years (Fig. S1, Supplementary Material) (3,4). One conceivable reason for this decline could be difficulties in development, since the technique by itself has many advantages.

LBF can help avoid food effects (5), reducing susceptibility to the patient’s prandial state and gastrointestinal physiology. By pre-dissolving the API in lipid excipients together with surfactants and co-solvents, the apparent solubility within the GI tract is less affected by the timing of administration. LBF has also been shown to increase the bioavailability of BCS (biopharmaceutics classification system) class II and IV drugs (5–8), possibly by circumventing the dissolution step in the intestine. However, several studies have shown that an API does not necessarily have to be fully dissolved within an LBF, but that co-administration with crystalline material can be sufficient to increase the amount of solubilized drug and bioavailability (9,10), in a phenomenon sometimes described as the “chasing principle” (11). This effect means that dissolution prior to administration might not be necessary for effective drug delivery via LBF. There are several hypotheses as to the mechanism(s) underlying this increased bioavailability, yet little conclusive proof. At the same time, there is a lack of useful *in vitro* methods for predicting the *in vivo* efficacy. Thus, it is difficult to optimize LBFs rationally and without conducting large numbers of animal experiments, which means that developing a drug product formulated with lipids might be less attractive than other advanced formulation techniques.

The current gold standard for evaluation of LBFs is the *in vitro* lipolysis assay in which formulations are dispersed in simulated intestinal fluid and subjected to digestion by pancreatic enzymes. This method could possibly be considered a more biorelevant drug release test than standard pharmacopeial dissolution methods (12). In the *in vitro* lipolysis assay, the amount of API solubilized – or available for absorption – is expected to correlate to the *in vivo* performance, because the APIs are typically highly permeable. However, when comparing LBFs of different compositions, the *in vitro in vivo* relationship (IVIVR) of drug release with bioavailability fails to reach even rank-order in many cases (9,13,14); the reader is advised to read the review by Feeney et al. (15) for further details. This observation suggests that the mechanisms of action for LBFs are not necessarily as simple as increasing apparent solubility within the intestinal media or supersaturation effects driving permeation. It should however be noted that supersaturation can be difficult to accurately determine (16).

Successful attempts at predicting the *in vivo* exposure from *in vitro* data have been made by simulations based on physiologically based pharmacokinetic (PBPK) models (17,18). When the absorption of drug and lipolysis products is taken into account, it is possible to predict the impact of the formulation on the fate of the API more accurately. These models require parameterization based on *in vivo* studies to simulate new *in vivo* data, as well as *in vitro* data as input. When this is available, PBPK simulations can provide very valuable mechanistic insights into the biopharmaceutical properties of the formulated drug. For initial screening of formulations however, PBPK simulations can present a significant challenge because of the input data and effort required to parameterize the models. A sufficiently biorelevant *in vitro* method – that incorporates simultaneous lipolysis and absorption – could offer the possibility of less time-consuming direct comparisons of formulations, without requiring preclinical pilot studies. The generated data can then be used to inform PBPK models and simulations at a later stage.

Recently, an *in vitro* method was published in which effectiveness of LBFs was gauged by adding simultaneous absorption via a Caco-2 monolayer to the *in vitro* lipolysis assay (19). This method has shown good IVIVR by assessing mass transfer across the monolayer (9,19), a promising step towards a more efficient development process for LBFs. However, cultured cells are not compatible with all formulations or digestive agents (20,21). Culturing and handling of cells also requires significant effort and expertise. To try to minimize these problems, we investigated the effectiveness of replacing the cell monolayer with an artificial (cell-free) lipid membrane. The method investigated in this study – implementing artificial membranes as the absorption compartment barrier – could potentially complement the cell-based method and allow formulations of APIs to be assayed more effectively, in particular those absorbed mainly by passive transcellular diffusion.

## Materials and Methods

### Materials

Acetonitrile (≥ 99.9%), methanol (99.9%), fenofibrate, warfarin, porcine pancreatin (8 x USP specifications), bovine serum albumin, dimethyl sulfoxide (DMSO, ≥ 99.9%) D-α-Tocopherol polyethylene glycol succinate (TPGS), hexadecane (anhydrous, 95%), Tris-maleate, 4-bromophenol boronic acid, olive oil, Kolliphor EL (macrogolglycerol ricinoleate), Kolliphor RH40 (macrogolglycerol hydroxystearate), Tween 85, and Carbitol (diethylene glycol monoethyl ether) were purchased from Merck (Darmstadt, Germany). Felodipine was kindly donated by Lundbeck Pharma (Valby, Denmark). Captex 355 and Capmul MCM EP (Abitec, Janesville, WI, USA) were kindly donated by Barentz (Odense, Denmark). Miglyol 812 N was obtained from IOI Oleo (Wittenberge, Germany). FaSSIF/FeSSIF/FaSSGF powder were bought from Biorelevant.com (Croydon, UK). Lucifer Yellow CH dilithium salt was obtained from Biotium (Fremont, CA, USA). Lecithin 20% soy PC extract was obtained from Avanti Polar Lipids (Alabaster, AL, USA). GIT-0 lipid solution and Acceptor Sink Buffer were purchased from Pion (Billerica, MA, USA). *N*-dodecane (≥ 99%) was obtained from Alfa Aesar (Lancashire, UK). Ethanol (99.5%, denatured with 0.4% isopropyl alcohol) was obtained from Solveco (Rosersberg, Sweden). All water used was of grade I from a Milli-Q lab water purification system (Merck).

### Artificial Membrane Preparation

Hexadecane membranes (HDMs) were prepared as previously described by Matsson et al. (22). In short, a 5% (*v*/v) solution of hexadecane in hexane was added to Transwell inserts (Corning, polycarbonate filter, 24 mm diameter, 10 μm thickness, 0.4 μm pore size with nominal pore density 1 × 10^8^ pores/cm^2^). The hexane was allowed to evaporate for at least one hour from the filter-immobilized hexadecane membrane (HDM). HDMs were hydrated with 10 mM phosphate buffer solution for 30 min before use, at 37°C in an orbital shaker at 400 rpm.

Based on data extracted from DS-PAMPA method (23) and the GIT-0 formulation (24), a generic membrane-forming solution – referred herein as lecithin-in-dodecane (LiDo) – was prepared by dissolving Avanti’s 20% Soy PC extract in a solution of 1.5% (*v*/v) absolute ethanol in *n-*dodecane, to a final concentration of 20% (*w*/*v*) lecithin. After overnight dissolution, the solution was centrifuged at 3220 *g* at 20°C for 20 min to remove undissolved material. The LiDo solution was then aliquoted and stored under argon at −18°C until use. This preparation corresponds functionally to the commercially available GIT-0 lipid solution (Fig. S2, Supplementary Material). Quantitative analysis showed less than 10% difference in relative abundance per phospholipid species between lab-prepared and commercially procured lipid solution. LiDo and GIT-0 solutions were thawed at room temperature overnight, and membranes were prepared 10 min prior to the start of experiments by coating either polycarbonate or PVDF filter supports (Millipore Immobilon-P, 0.45 μm pore size, thickness 100–145 μm) with 16.2 μl of lipid solution per cm^2^ of filter.

### Conventional *In Vitro* Lipolysis Method

Lipolysis was performed as previously described by Alskär et al., using the Metrohm Titrando equipment with automated pH-titration and overhead stirring of the medium with an impeller (25). In short, 1.66 g of formulation was weighed into a jacketed glass vessel kept at 37°C, followed by addition of 53.3 ml digestion medium. The digestion medium was prepared by dissolving FaSSIF/FeSSIF/FaSSGF powder (2.24 g/l, obtaining 3 mM taurocholate and 0.75 mM lecithin) in lipolysis buffer (pH 6.5, containing 2 mM Tris-maleate, 150 mM NaCl, and 1.4 mM CaCl_2_). The mixture was dispersed at 450 rpm for 10 min and if necessary, manually adjusted to pH 6.5 using 0.1 M NaOH solution. After 10 min of dispersion, 5.92 ml porcine pancreatin extract was added to initiate digestion. The extract was prepared by dispersing 1.6 g porcine pancreatin powder in 8 ml cold lipolysis buffer, followed by centrifugation at 2690 *g* for 15 min (5°C) and extracting the supernatant (6600 TBU/ml, corresponding to ~4000 USP units/ml (26)). A 0.6 M NaOH solution was used as titrant to keep pH stable at 6.5 by autotitration during the digestions. To estimate the total extent of digestion, the amount of titrant required to raise the pH to 9.0 and ionize all of the released free fatty acids was measured. For this measurement, a lipolysis of blank digestion medium was used as reference to exclude digestion medium effects from the required titrant volume.

### Integrity Assay with Static Digest Matrix

**Fig. 1:**
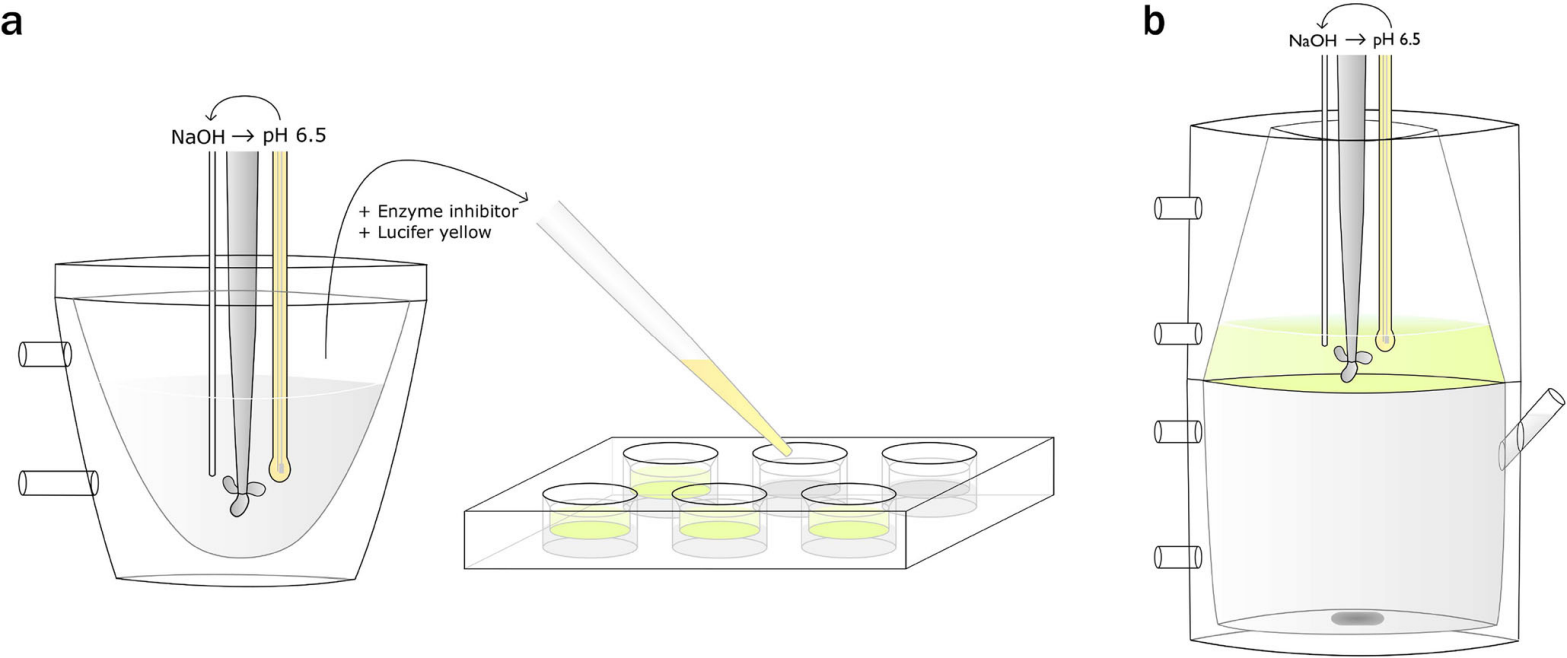
Graphical overview of experimental procedure. (**a**) Integrity assay static digest matrix. Dispersion and digestion of a lipid-based formulation was performed in single-compartment *in vitro* lipolysis. Samples were taken at different points and digestion inhibited with 4-bromophenol boronic acid. Lucifer Yellow (LY, 10 mM in DMSO) was added to a concentration of 10 μM before the sample was added to filter plate-inserts treated with artificial membranes. The permeation of LY was then measured by fluorescence detection. For integrity assays under dynamic digestion conditions, dispersion and digestion was performed *in situ* in the inserts. (**b**) *In vitro* lipolysis and permeation assay of loaded lipid-based formulations. Formulations loaded with fenofibrate were dispersed in the top (donor) compartment and separated from the bottom (receiver) compartment by a PVDF filter treated with LiDo to form an artificial membrane.

Six samples of 4 ml were taken during dispersion and digestion phases of single-compartment *in vitro* lipolysis (Fig. 1a) of a formulation over a period of 40 min. The formulation was of type IIIB-MC (medium chain length triglyceride component, 8–10 carbons) according to the Lipid Formulation Classification System (LFCS) (27), see Table I for composition. Lipolysis was immediately inhibited in the samples through addition of 0.5 M solution of 4-bromophenol boronic acid in methanol (5 μl/ml of sample). To visualize membrane integrity, Lucifer Yellow (LY) in DMSO solution was added to each sample to a final LY concentration of 10 μM and 0.1% DMSO. LY is highly hydrophilic; its permeation through intact lipid membranes should therefore be negligible. The sample tubes were then vortexed, and shaken at 450 rpm at 37°C until further use.

**Table I:** Compositions of Formulations and Properties of Excipients Used

LBF type^a^	Digestible lipid excipients	% (*w*/w)	C:D^b^	Surfactant excipients	% (w/w)	HLB^c^	Co-solvent excipients	% (w/w)
IIIB-MC	Captex 355	12.5	8:0, 10:0	Kolliphor EL	50	12–14	Carbitol	25
	Capmul MCM EP	12.5	8:0, 10:0					
IIIA-MC	Miglyol 812 N	40	8:0, 10:0	Tween 85	40	11	–	–
				Kolliphor RH40	20	14–16		
IIIA-LC	Olive oil	40	18:1–2, 16:0	Tween 85	40	11	–	–
				Kolliphor RH40	20	14–16		
IV	–	–	–	Tween 85	67	11	–	–
				Kolliphor RH40	33	14–16		

^a^Lipid-based formulation (LBF) type based on the Lipid Formulation Classification System (27) and lipid chain length, as either medium chain (MC) or long chain (LC). ^b^Number of carbons (C) and unsaturations (D) in the acyl chains of the respective digestible lipids comprising the formulation. ^c^Hydrophilic-lipophilic balance values (HLB) of non-ionic surfactants according to manufacturer information

Digestion samples were dispensed (1.2 ml) into the donor compartments of Transwell inserts treated to form artificial membranes. Negative controls comprised only blank digestion medium or lipolysis buffer (both containing LY, 10 μM) in the donor compartment. Positive controls were untreated inserts composed of naked polycarbonate filters, or GIT-0 solution added to pre-wet filters (disrupts membrane formation). The receiver compartments contained 2 ml of either plain phosphate buffer (PB, 10 mM), Acceptor Sink Buffer (20 mM HEPES, 1% *w*/*v* SDS), or PB supplemented with 4% *w*/w bovine serum albumin (BSA). All receiver buffers were adjusted to pH 7.40.

From examination of mass transfer profiles, membrane damage was evaluated by Area Under the Curve (AUC) values of LY mass transfer over time. The criteria were that an LY AUC > 10 nmol min cm^−2^ was considered to indicate major membrane disruption, while an LY AUC < 1 nmol min cm^−2^ was considered to indicate an intact membrane.

### Integrity Assay under Dynamic Digestion Conditions

HDM, GIT-0 and LiDo membranes were subjected to *in situ* lipolysis of the IIIB-MC LBF in a 6-well culture plate format. Transwell inserts (diameter of 24 mm) were used for polycarbonate filters, and inserts were made in-house to hold the PVDF filters (diameter of 25 mm). For these lipolysis experiments, a modified digestion medium with greater buffering capacity was used. The digestion medium contained 200 mM Tris-maleate in these experiments to maintain pH within 0.3 units of the initial value of the lipolysis buffer (pH 6.5) over one hour of digestion (Table S2, Supplementary Material), since no autotitration could be used in the setup. LBF was weighed into a glass tube, and 10 ml of this modified digestion medium spiked with 10.3 μl of LY in DMSO solution (10 mM) was added and vortexed. To the insert donor compartments, 1.35 ml of this mixture was then immediately added, and the plates were placed in an orbital shaker for 10 min (400 rpm, 37°C). This step was intended to mimic the dispersion phase of an *in vitro* lipolysis assay, after which digestion was initiated through addition of 148 μl of porcine pancreatin extract per insert (final activity ~750 TBU/ml).

GIT-0 and LiDo membranes were also evaluated in a large-scale (75 mm diameter) in-house developed lipolysis-permeation device named “ENA” (19) in order to explore whether these two lipid mixtures with theoretically similar compositions and production methods also yielded similar results. These experiments were performed as for a conventional single-compartment *in vitro* lipolysis assay, but with LBF and LY (10 μM) dispensed into the digestion medium (2 mM Tris-maleate) with stirring. The receiver compartment contained 10 mM phosphate buffered saline (140 mM NaCl, 2.7 mM KCL, pH 7.4) supplemented with 4% (*w*/w) BSA for all integrity assays under dynamic conditions.

### *In Vitro* Lipolysis and Permeation Assay of Loaded LBFs

Three formulations representing LFCS types IIIA-MC, IIIA-LC (long chain fatty acid component), and type IV (Table I) were assayed for their performance using artificial membranes in the ENA device. No more than one week before the assay, excipients were heated to 37°C before being weighed into a glass vial. The vial was capped with argon, vortexed, and then shaken overnight (450 rpm, 37°C). The formulations were then loaded with fenofibrate (80 mg/g) in the same manner, and shaken until complete dissolution. This loading corresponded to 55.6, 82.8, and 76.6% of equilibrium solubility at 37°C for LBF types IIIA-MC, IIIA-LC, and IV, respectively (14).

The loaded formulations were weighed separately and then dispensed into ENA donor compartments (Fig. 1b) that had been prefilled with digestion medium spiked with LY to 10 μM (0.1% DMSO). Samples were taken from both compartments during dispersion (0–10 min) and digestion (10–100 min). Samples from the donor compartment were immediately filtered through a hydrophilic nylon syringe filter (Whatman PuraDisc 13, pore size 0.1 μm) to separate precipitates and oil droplets from fenofibrate in free solution and in colloidal aggregates, using the same procedures as those described in Juenemann et al. and Stillhart et al. (28,29). The syringe filters were then flushed through with room temperature acetonitrile to recover precipitated fenofibrate. It was not possible to filter some early samples (5–20 min) of type IIIA-LC formulation digestion due to high backpressure. Phases were instead separated through centrifugation (37°C, 21,000 *g* for 15 min) for these samples. Receiver compartment samples were then quantified using UPLC-MS, and donor samples using HPLC-UV.

### Sample Analysis

LY presence was detected using fluorescence (Spark or Safire^2^ plate readers, Tecan, Austria) at 428 and 536 nm wavelength for excitation and emission, respectively. Samples were diluted 1:2 in ice-cold acetonitrile and centrifuged (4°C, 2465 *g* for 20 min) to precipitate protein content.

Fenofibrate was analyzed using a UV-DAD coupled HPLC (1290 Infinity, Agilent Technologies) with a Zorbax Eclipse XDB-C18 column (4.6 × 100 mm, Agilent Technologies) kept at 40°C (injection volume 20 μl). The mobile phase consisted of sodium acetate buffer (25 mM, pH 5.0) in acetonitrile solution (2:8 *v*/v) with isocratic flow (1 ml/min). UV absorbance was monitored at a wavelength of 287 nm. The retention time was 3.32 min. Sample preparation consisted of 100x dilution in mobile phase and a centrifugation step (21,000 *g*, 15 min, 25°C) to purify the matrix.

UPLC-MS analysis was performed using a Xevo TQ MS coupled Acquity UPLC system (Waters, Milford, MA) with a BEH C18 column (2.1 × 50 mm, 1.7 μm, Waters). The mobile phase consisted of 5% acetonitrile and 0.1% formic acid in water (solvent A), and 0.1% formic acid in acetonitrile (solvent B). Gradient elution at a constant flow rate of 0.8 ml/min was used. Mobile phase A was decreased linearly (95 to 0%) from 0.2 to 0.65 min, followed by a constant flow for 0.15 min, and then a linear increase back to 95% A at 0.8 min until the end of the run (1 min, injection volume 10 μL). The column oven and auto-sampler tray temperature were set at 60°C and 10°C respectively.

The mass spectrometer was operated in positive electrospray mode for fenofibrate and fenofibric acid, and in negative mode for warfarin (internal standard of the analytics). The retention times of these compounds were 0.89, 0.79, and 0.76 min, respectively. Precursor-product ion pairs followed were: (i) m/z 361 → 233 (cone voltage 20 and collision energy 16 V) for fenofibrate, (ii) m/z 319 → 139 (cone voltage 20 and collision energy 32 V) for fenofibric acid, and (iii) m/z 309 → 163 (cone voltage 22 and collision energy 14 V) for warfarin. Data acquisition and peak integration were performed with MassLynx software (Waters). Sample preparation consisted of dilution in receiver buffer (1x, 10x, and 100x), followed by dilution 1:2 in ice-cold warfarin in acetonitrile solution (100 nM), and a centrifugation step (4°C, 2465 *g* for 20 min) to precipitate albumin from the matrix. Fenofibric acid was not used in the study, but merely controlled for during analysis as routine control of API degradation during lipolysis and sample storage. As no fenofibric acid could be detected in any samples, there was no attempt at quantification.

### Data Analysis

Data are presented as mean values with standard deviation (*n* = 3, unless otherwise specified). Statistical analysis was performed in GraphPad Prism 7 (GraphPad Software, USA) using Student’s t test to evaluate differences between two groups, or a one-way ANOVA followed by a Tukey’s multiple comparison analysis test, to compare differences for more than two groups. Holm-Sidak’s multiple comparisons test was used when comparing against a control, as Tukey’s test does not support this. Two-way ANOVA was used to compare groups with two differing factors (formulation and barrier model). *P*-values <0.05 were considered statistically significant. Area under the curve (AUC) was calculated via a Python (version 3.6.5) script by fitting data to cubic splines using scipy.interpolate.CubicSpline and integrated using scipy.integrate.IntegrateQuad (SciPy version 1.1.0).

Formulation rankings between different assays was compared by normalizing AUC values of either donor, receiver, or plasma concentrations. The normalization was done by converting AUC values into percentages of the group sum, where 100% was defined as the sum of mean values in each respective group. The six groups were as follows: cell-free system donor samples with filter separation of phases (ENA/LiDo, this work); cell-free receiver samples (ENA/LiDo, this work); cell-based system donor samples with centrifugation separation of phases (ENA/Caco-2) (19); cell-based receiver samples (ENA/Caco-2) (19); single-compartment *in vitro* lipolysis samples with ultracentrifugation separation of phases (14); and *in vivo* plasma samples from pigs (14).

Apparent permeability coefficients (P_app_) were calculated using the Solver tool in Microsoft Excel 2016 by non-linear least-squares regression of Eq. 1 (two-way flux model), where the assumption of sink-condition is not required (30).

1 $$ {C}_R(t)=\frac{M}{V_D+{V}_R}+\left({C}_{R,0}-\frac{M}{V_D+{V}_R}\right){e}^{-{P}_{app}\ast A\ast \left(\frac{1}{V_D}+\frac{1}{V_R}\right)\ast t} $$

where *C*_*R*_ is the concentration (nmol/cm^3^) in the receiver compartment at time *t* (sec). *M* is the total amount (nmol) of analyte in the system available for permeation. *C*_*R,0*_ is the concentration of analyte in receiver compartment at *t* = 0. *V*_*D*_ is the total volume (cm^3^) of the donor compartment, *V*_*R*_ is the total volume (cm^3^) of the receiver compartment, and *A* is the total area (cm^2^) available for permeation between compartments. For the data analyzed in this work, *M* was assumed as the summed amount detected in aqueous fractions and receiver at each time point. *M* at *t* = 0 was assumed as the total amount added to the system.

Equation 1 approximates the system as being composed of two compartments, a donor and a receiver, with the lipid or cell membrane having little impact other than a rate-limiting effect that is described by P_app_. However, depending on the analyte, the membrane can act as a discrete compartment and retain some of the permeating compound. This can be a large fraction of the total amount if the analyte has high affinity for the membrane constituents, and the receiver compartment is not a sufficiently effective sink. In cell-based systems, retention may also be a result of lysosomal trapping, which mainly basic compounds are at risk for due to the pH-driven entrapment (1,31). If the membrane retention is known, this fraction can be subtracted from the total amount of analyte in the system and the permeability coefficient adjusted accordingly. Furthermore, when comparing the permeability of a compound assayed in systems with differing filter support materials, the nominal porosity of the filter (ε) can affect P_app_. The nominal porosity (ε) of the filter is approximately equal to the area of each pore (pore size), multiplied by the number of pores, and divided by the total area of the filter. A nominal porosity lower than unity therefore decreases the uncorrected P_app_, as the available area for permeation is decreased. Multiplying the geometric area of the filter with the nominal porosity therefore describes the area that is available for permeation more accurately (Eq. 2).

2 $$ {C}_R(t)=\frac{M\ast \left(1-\mathcal{R}\right)}{V_D+{V}_R}+\left({C}_{R,0}-\frac{M\ast \left(1-\mathcal{R}\right)}{V_{\mathrm{D}}+{V}_R}\right){e}^{-{P}_{app}\ast A\ast \varepsilon \ast \left(\frac{1}{V_D}+\frac{1}{V_R}\right)\ast t} $$

where ℛ is the membrane retention factor (mol%), which herein was approximated from mass balance (total analyte recovered subtracted from amount added at start of experiment) at each data point used for fitting. According to the manufacturer, the nominal porosity is 0.70 for the Immobilon-P PVDF (0.45 μm pore size) filter used in this study. For the Transwell polycarbonate filters used in the Caco-2 study (19), the nominal porosity input was 0.16 in accordance with Matsson et al. (22).

## Results

### Integrity Assay with Static Digest Matrix

**Fig. 2:**
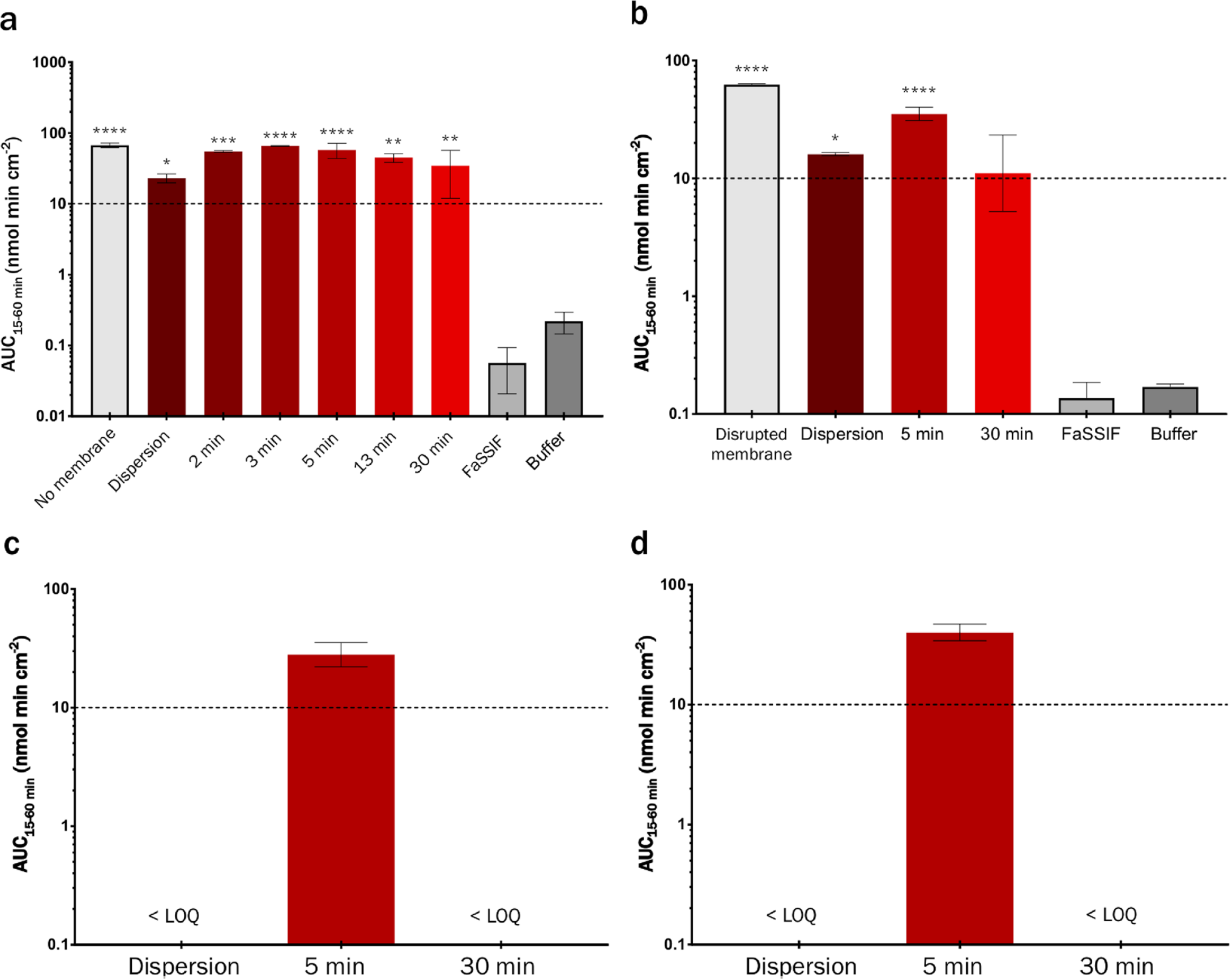
Mass transfer of permeation marker LY (AUC values, 15–60 min) over artificial membranes subjected to varying stages of LBF digestion (inhibited enzymes). Negative controls comprised LY in digestion medium (FaSSIF) or lipolysis buffer (used as control for significance testing). Positive controls comprised LY in FaSSIF on either naked polycarbonate filters or GIT-0 membranes disrupted by applying the solution to pre-wet filters. Values above the dashed line indicates a major loss of membrane integrity. (**a**) Hexadecane membranes (HDM), (**b**) GIT-0 membranes (soy lecithin in *n-*dodecane) with phosphate buffer (PB, 10 mM) in the receiver compartment, (**c**) GIT-0 membranes with Acceptor Sink Buffer (containing 20 mM HEPES and 1% *w*/*v* SDS) in receiver compartment, and (**d**) GIT-0 membranes with PB supplemented with 4% (*w*/w) bovine serum albumin (BSA) in receiver compartment.

Samples taken from *in vitro* lipolysis at −5, 2, 3, 5, 13 and 30 min of digestion corresponded to mean values of 0, 18, 35, 46, 56, and 65% (*n* = 5) of complete digestion. Membrane integrity was maintained for one hour of exposure to digested media for 8 out of 21 conditions (Fig. 2). Four of the eight conditions with maintained membrane integrity were negative controls. The remaining four were the samples taken at −5 and 30 min of lipolysis (0 and 65% digestion) and applied to GIT-0 membranes with phosphate buffer ± BSA in the receiver (Fig. 2c–d). After two hours of exposure, only membranes exposed to negative controls and the two LBF samples applied to GIT-0 with phosphate buffer (no BSA) in receiver remained intact (Fig. S3, Supplementary Material), out of which only one had been subjected to digestion (30 min sample).

For HDMs subjected to LBF-containing media (Fig. 1a), only the dispersion-phase and late-stage digestion (30 min) samples were significantly differentiated from positive controls, but all samples were significantly different from negative controls. Based on these results, we concluded that HDMs were incompatible with triglyceride-containing LBFs. GIT-0 membranes (Fig. 2b–d) were more resilient, exhibiting mass transfers of LY that were significantly lower than the positive controls for all conditions containing LBFs. The composition of the receiver buffer was however observed to affect mass transfer. The Acceptor Sink Buffer, containing 1% (*w*/*v*) anionic surfactant (SDS), resulted in loss of integrity of GIT-0 membranes when exposed to LBF-containing media (Fig. 2b). Supplementation with 4% (*w*/w) protein (BSA) instead of SDS did not cause significantly different mass transfer of LY across GIT-0 (Fig. 2c) compared to plain phosphate buffer in the receiver (Fig. 2d). No lipid-polycarbonate filter combination could withstand the mid-stage digestion (5 min) samples for more than half an hour. These results deemed polycarbonate filter supports inappropriate for assays involving digestion and absorption.

### Integrity Assay under Dynamic Digestion Conditions

**Fig. 3:**
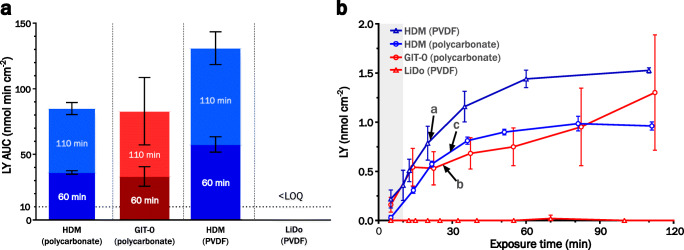
(**a**) LY exposure in receiver with HDM (blue) or LiDo membranes (red), subjected to digestion of a type IIIB medium-chain LBF by porcine pancreatin. Filter support material was either polycarbonate or PVDF (polyvinylidene difluoride). Area under the curve (AUC) values are calculated on exposure time of either 60 min (darker) or 110 min (lighter). Bars reaching above the horizontal dotted line indicate a major loss of integrity. (**b**) Corresponding LY mass transfer curves for HDM (blue), LiDo or GIT-0 (red), on polycarbonate (circles) or PVDF (triangles) filter support. Arrows point to the time points in each system at which AUC ≈ 10 nmol min cm^−2^, corresponding to a) 21, b) 26, and c) 29 min of LY exposure. The gray shaded field indicates the time before addition of digestion agent (10 min after experiment start).

Similar to what was observed with the static digestion matrix, polycarbonate-filter supported lipid membranes did not maintain an acceptable level of integrity for any suitable experiment length when porcine pancreatin was used as the digestive agent (Fig. 3a–b). In the dynamic setting, LiDo membranes could not be significantly differentiated from HDMs when applied to polycarbonate membranes and exposed to porcine pancreatin.

HDMs using PVDF filters for support showed a significantly greater mass transfer of LY during the digestion phase than polycarbonate-supported HDMs. Contrarily, LiDo on PVDF did not allow detectable levels of LY through the membrane for up to two hours, although an increase in donor compartment pH was observed after circa 90 min of digestion (Fig. S4, Supplementary Material). This pH rise can be regarded as an *in situ* marker for membrane integrity loss (19).

### *In Vitro* Lipolysis and Permeation Assay of Loaded LBFs

**Fig. 4:**
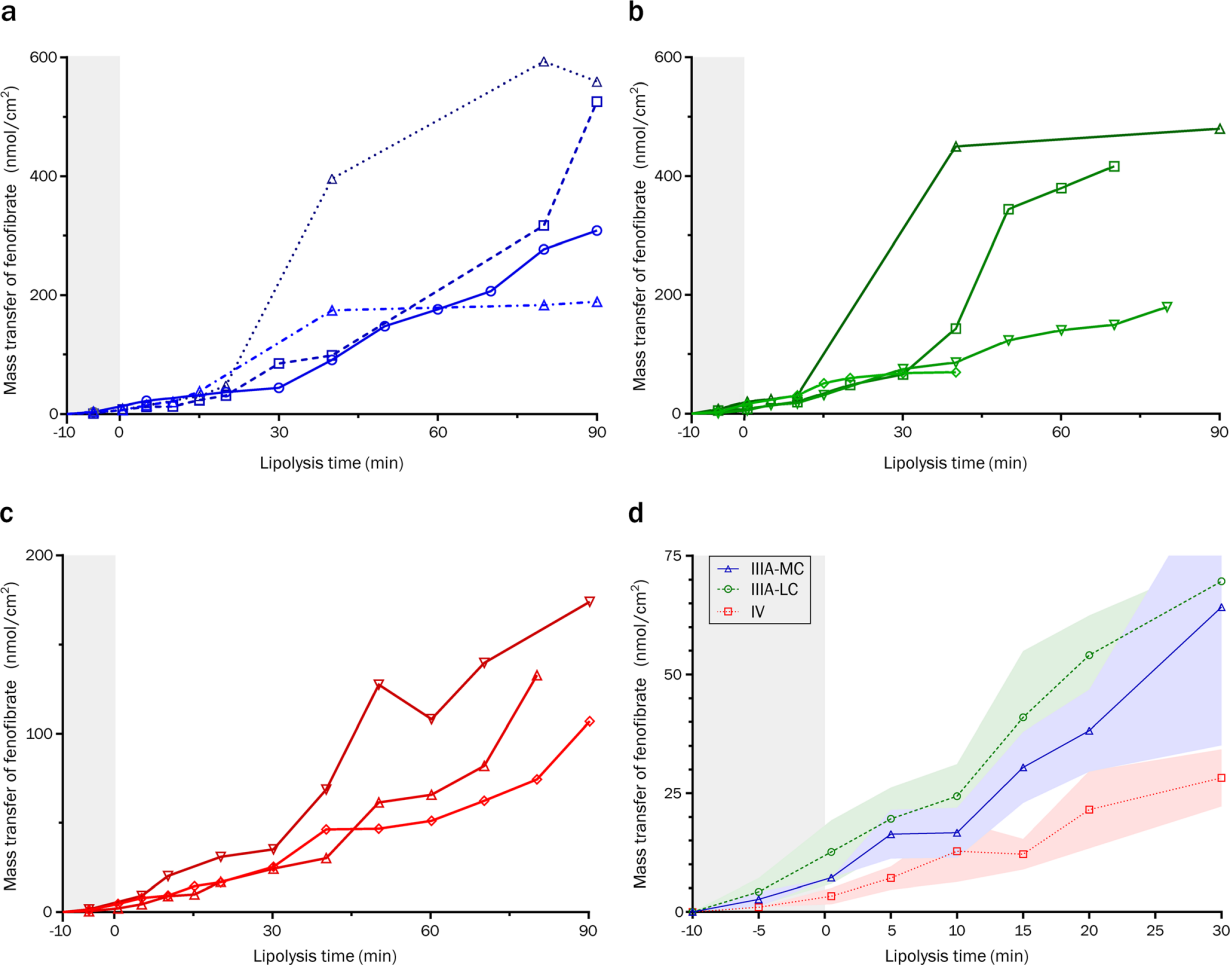
Mass transfer through LiDo membranes with PVDF filter support (in ENA) of fenofibrate loaded in different lipid-based formulations. Gray shaded areas indicates the dispersion phase (10 min) before addition of porcine pancreatin (t = 0). (**a–c**) Individual replicates of data collected from −5 to 90 min for different LBF types: (**a**) IIIA-MC; (**b**) IIIA-LC; (**c**) type IV. (**d**) Collated mass transfer data (−5 to 30 min) for type IIIA-MC (blue triangles), IIIA-LC (green circles), and IV (red squares). The colored shaded areas show standard deviations. Abbreviations: medium chain (MC); long chain (LC)

Because of the high integrity of the LiDo/PVDF combination, it was used in the ENA device to further study whether and how this artificial membrane could identify promising formulations. Three different formulations loaded with fenofibrate were studied. Samples were taken from both compartments during 90 min of digestion. In a large proportion of the experiments, the LBFs containing digestible lipids (types IIIA-MC and LC) induced a sharp increase in mass transfer of fenofibrate after 30–60 min of digestion (Fig. 4a–c). The data from samples taken after 30 min of lipolysis were therefore excluded from comparisons (Fig. 4d). No corroborating increase in the mass transfer of the hydrophilic marker for integrity (LY) was evident.

**Fig. 5:**
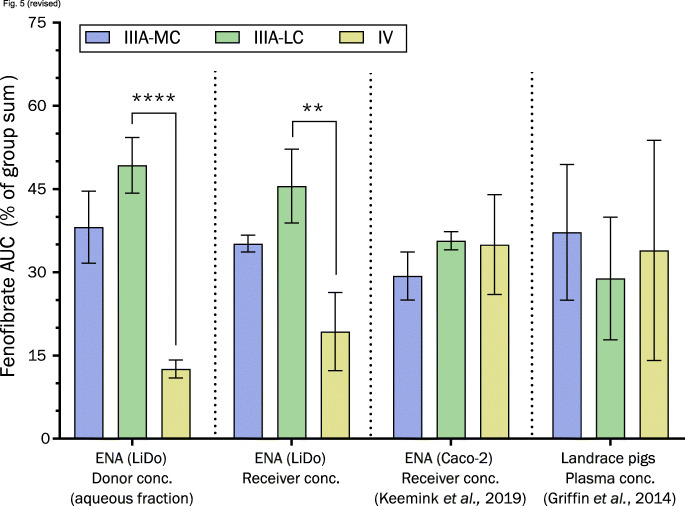
Comparison of fenofibrate loaded LBFs in various models. LBFs were of type IIIA-MC (blue), IIIA-LC (green), and IV (yellow). Normalized AUC values (percentage of the sum of AUC means within each group) for comparison of ranking between groups. “Donor conc.” is the concentration of fenofibrate, either in free solution or in colloidal aggregates within the donor matrix. Values derived from ENA are 10 min of dispersion, followed by 30 or 60 min of digestion in LiDo (this work) and Caco-2 models (19), respectively. Plasma AUC in pigs is derived from 0 to 24 h after administration (14). Abbreviations: medium chain (MC); long chain (LC)

The type IV formulation resulted in significantly lower concentrations of solubilized fenofibrate than types IIIA-MC or IIIA-LC (Fig. 5), which agrees with previously conducted *in vitro* lipolysis without absorption components (Fig. S5a–b, Supplementary Material) (14). Statistically significant differences of fenofibrate mass transfer between formulation types IV and IIIA-LC, and between IV and IIIA-MC, were also found. The formulation rank-order based on permeated fenofibrate was the same as the rank-order based on solubilized fenofibrate in the donor compartment.

**Fig. 6:**
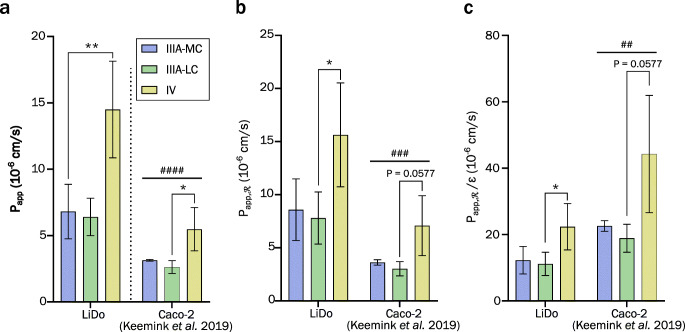
Calculated apparent permeability coefficients (P_app_) from ENA (LiDo and Caco-2) data from lipolysis-permeation assays of fenofibrate loaded LBFs of type IIIA-MC (blue), IIIA-LC (green), and IV (yellow). Pound signs indicate significance level between the barrier models as given by two-way ANOVA. (**a**) Unadjusted P_app_-values from Eq. 1 (**b**) Coefficients corrected only for mass balance as proxy for membrane retention (ℛ). (**c**) Coefficients adjusted for ℛ and filter porosity (ε) according to Eq. 2. Abbreviations: medium chain (MC); long chain (LC).

Observed apparent permeability coefficients (P_app_) indicated a high degree of similarity between type IIIA-MC and IIIA-LC LBFs (Table II). The type IV formulation was also in this regard different from the triglyceride-containing formulations, showing a significantly higher P_app_ (Fig. 6a). Re-analysis of data from previously conducted experiments with Caco-2 monolayers (19) revealed a similar relationship among the formulations, as well as a slight but significant difference in the magnitude of permeability between Caco-2 and LiDo models. The ratio between Caco-2 and LiDo permeability was on average 0.42. Correcting for mass balance to calculate retention adjusted values (Eq. 2) did not significantly affect the apparent relative difference between Caco-2 and LiDo (Fig. 6b). However, after adjusting for filter porosity (ε), the relationship between the two model systems was inverted and Caco-2 permeability on average 1.8-fold higher than that of LiDo (Fig. 6c).

**Table II:** Apparent permeability coefficients (P_app_, in 10^−6^ cm/s) from mass transfer data of fenofibrate loaded in different LBFs, assayed with LiDo and Caco-2 models in the ENA system. Data presented as mean ± standard deviation (*n* = 3–4)

	Uncorrected^a^		Corrected for ℛ^b^		Corrected for ℛ and ε^b^	
**Formulation**	**LiDo**	**Caco-2**^**c**^	**LiDo**	**Caco-2**^**c**^	**LiDo**	**Caco-2**^**c**^
IIIA-MC	6.8 ± 2.1	3.1 ± 0.1	8.6 ± 2.9	3.6 ± 0.3	12.3 ± 4.1	22.6 ± 1.6
IIIA-LC	6.4 ± 1.4	2.6 ± 0.5	7.8 ± 2.5	3.0 ± 0.7	11.1 ± 3.5	18.9 ± 4.2
IV	14.5 ± 3.6	5.5 ± 1.6	15.6 ± 4.9	7.1 ± 2.8	22.3 ± 7.0	44.3 ± 17.7

^a^P_app_ from Eq. 1. ^b^P_app_ adjusted for membrane retention (ℛ) or ℛ and filter porosity (ε) (Eq. 2). ^c^Data re-analyzed from Keemink et al. (19)

## Discussion

During the initial characterization process, two artificial membrane types were selected due to their ease of use, low cost, and extensive permeability characterization (1). Filters impregnated with hexadecane (HDMs) are among the simplest and most inexpensive artificial membranes available and have been used for permeability assessment previously (1,22,32). These are typically prepared on polycarbonate filter supports, where correct choice of pore size is important for maintaining membrane integrity (22). The interaction between the lipid membrane and its support material is clearly important for the artificial membrane to maintain barrier properties.

We initially assumed that HDMs could withstand contact with a lipolysis process, as hexadecane lacks ester groups that can be digested by pancreatic lipase (33). In this study, however, it became clear that HDMs could not retain their integrity for longer than 10 min during active digestion of LBFs by porcine pancreatin. Additionally, we showed that a dispersed triglyceride-containing LBF alone is sufficient to compromise HDM integrity. Switching to a more hydrophobic backing substrate (PVDF) did not improve the robustness of the model.

The GIT-0 model membrane is more complex in composition than HDM, due to the soy lecithin dissolved in *n-*dodecane. It was developed to be easy to use while allowing permeabilities for a wide range of drug compounds to be accurately predicted, and is marketed for measuring permeability coefficients (PAMPA) or use in drug release and absorption assays (μFlux/MacroFlux) (24,34,35). It therefore seemed a reasonable candidate for simultaneous lipolysis-permeation assays.

Unlike HDMs, which are typically prepared by solvent evaporation on polycarbonate substrate, GIT-0 is dispensed on top of a hydrophobic PVDF substrate and used within 10 min. Prepared HDMs that are ready for use appear dry, while GIT-0 membranes ready for use appear liquescent. At first glance, GIT-0 membranes might not seem resilient in a well-stirred system, but the results indicate that they are more resilient than HDMs. The interaction between the membrane-forming solution and the filter substrate material was also important. GIT-0 was ineffective on polycarbonate, but effective on PVDF. Furthermore, the in-house solution referred to as LiDo in this work, could not be distinguished from the commercially available GIT-0 solution in either function or composition (Fig. S2, Supplementary Material). For these reasons, LiDo applied to PVDF filter support was chosen as the preferred artificial membrane model for all further experiments.

To validate the model, three representative LBFs were selected for comparison. *In vitro* solubilized fractions, and absorbed/permeated fractions of fenofibrate among three different studies were compared: LiDo based lipolysis-permeation assay (this work), conventional *in vitro* lipolysis and *in vivo* plasma profiles (14), as well as the Caco-2 based lipolysis-permeation assay (19). For *in vitro* solubilized fractions, the ranking of the formulations is the same, despite some differences in experimental protocol (phase separation methods, digestive agents, media composition, and pH). However, re-analyzing data from (19) showed significantly higher relative aqueous levels of fenofibrate with the type IV formulation in the Caco-2 based lipolysis-permeation assay (Fig. S5, Supplementary Material), compared the LiDo based assay used in this work and the conventional *in vitro* lipolysis assay (14).

As shown in the Results, an inflection point in the flux of fenofibrate was seen in several experiments after approximately 30 min of digestion. It is likely that the LiDo membranes eventually suffer some damage from the formulations or their digestion products, leading to an increased mass transfer of the model drug. To judge from the lack of pH change in the donor medium or noticeable LY mass transfer to the receiver, the damage does not seem to be extensive enough to form aqueous pores in the membrane. The structure of LiDo or GIT-0 membranes is not known, however Assmus et al. proposed a model structure for a similar system (9% *w*/w egg lecithin in *n*-dodecane) based on NMR experiments (36). In this model, the dodecane envelops the PVDF filter support and the amphiphilic lecithin constituents form monolayers on either side, bridging the oil-water interfaces. Reverse micelles are proposed as carriers of hydrophilic compounds within and across the artificial membrane, whereas lipophilic compounds can diffuse across through the dodecane. Insertion of free fatty acids or lysophospholipids generated by digestion might alter the spontaneous curvature of the monolayer at the oil-water interface. Conceivably, this could lead to formation of narrow aqueous pores with net negative surface charge. As LY carries permanent negative charge, whereas fenofibrate is uncharged, electrostatic repulsion might explain the lack of a simultaneous inflection point for LY. The absence of spontaneous pH increase in the donor medium is more difficult to explain if aqueous pores are indeed formed within this period. However, partially formed pores or indentations would increase the apparent permeability of fenofibrate with decreasing height of the membrane.

With the exclusion of data measured at time points longer than 30 min, significant fenofibrate mass transfer differences between the formulations were still observed in the LiDo-based *in vitro* model. These results are at odds with those of Keemink et al. (19) and Griffin et al. (14) who assayed the formulations utilizing Caco-2 cell and landrace pig models, respectively. A difference can also be seen when comparing rank-order of the formulations between different models (Fig. 5), which indicates that the LiDo-based *in vitro* model with porcine pancreatin is more similar to the conventional single-compartment *in vitro* lipolysis model than the assays utilizing living tissue.

The observed effect of the formulations on the apparent permeability of fenofibrate do however indicate some underlying similarity between the LiDo and Caco-2 based models. After applying corrections for membrane retention and filter porosity, the permeability in the Caco-2 model was on average higher than that in the LiDo model. The height of the excess lipid on either side of the filter can be calculated from the volume added and the geometric properties of the filter in accordance with Nielsen & Avdeef (39). For the setting applied in this study, this results in a total height of 192–206 μm for LiDo membrane and PVDF filter through which a molecule must diffuse between donor and receiver compartments, excluding unstirred water layers. The filter height is approximately 100–145 μm for the PVDF filters in this study, and 10–20 μm for the polycarbonate Transwell filters used in the Caco-2 study (19) according to manufacturers. The height of Caco-2 cells can vary between 11 and 33 μm depending on the culturing protocol that is used and the source of the cells (37,38). This gives a total height or diffusion distance of 21–42 μm for a Caco-2 monolayer. The ratio between these distances given by each model is 4–9. As seen from the corrected permeability coefficients in Table II, the observed ratio (1.8) is approximately half of what could be expected from an approach purely based on the diffusion distance. While a more detailed analysis might yield somewhat different results, differences in the number of partitioning steps could in part explain the discrepancy.

Formulation ranking based on apparent permeability was inversely correlated with that of the solubilized concentrations over time in both models, P_app_ ranking by LBF type: IV > IIIA-MC ≈ IIIA-LC (Fig. 6a–c). If the solubilized drug concentration were the sole governing factor of mass transfer, P_app_ would be the same with all formulations. As the free fraction of fenofibrate is unknown in the tested systems, the true apparent permeability cannot be determined. Nevertheless, the similarity in ranking between the two models, based on P_app_, strengthens the idea that LiDo is functionally similar to Caco-2 in terms of passive permeability of fenofibrate, even under lipolysis conditions. Thus, LiDo cannot be discounted as a relevant lipid support to use for lipolysis-permeation assays, but the underlying issue causing relative mass transfer differences between the formulations tested here must be identified and remedied.

The simultaneous lipolysis-permeation assay with an artificial membrane was to our knowledge first attempted successfully in the work of Bibi et al. (40). They investigated the use of Permeapad, a phospholipid-based solid membrane sandwiched in-between two support sheets. In the study, an impressive durability of the membrane was demonstrated, where integrity could be retained for at least 24 h. However, the low mass transfer rates permitted by the Permeapad membrane is not ideal for assaying a system where thermodynamic stability is low such as a lipid-digestion driven, supersaturated system. It would require long assay times to reach quantifiable concentrations in the receiver compartment with the risk that rapid changes in saturation levels are missed. In contrast, the LiDo-based model membrane permits a more *in vivo*-like flux of the model drug fenofibrate. Under comparable conditions, the flux of fenofibrate over LiDo was on average 334-fold higher than the flux of cinnarizine over Permeapad. To summarize, Permeapad certainly displays impressive resilience to lipolysis conditions, and is certainly valuable for easy to use and cost-effective assays of thermodynamically stable systems. LiDo appears more suitable for fast assays of systems with rapid kinetics such as those with low thermodynamic stability.

In terms of realistic flux, the LiDo membrane permitted a similar flux as Caco-2. Based on the expected permeability of fenofibrate in human small intestine (17), the flux could be expected to be 10-fold higher *in vivo*. Dispersed LBFs are in most cases thermodynamically unstable systems, especially when submitted to lipolysis. The fraction absorbed is therefore a function of precipitation *versus* absorption of the API *versus* the residence time of the drug in the intestinal tract. In a well-stirred system, the precipitated API might redissolve and be absorbed if the membrane flux is sufficient. An artificially low flux will not provide sufficient biorelevance to produce meaningful results that capture the solubility-permeability interplay. The high flux over LiDo in the ENA system is likely due to the liquescent membrane structure, relatively large surface area and efficient stirring.

Fenofibrate is a highly permeable compound (17,41,42) and to our knowledge not actively transported. The observed differences in mass transfer ranking between the Caco-2 and LiDo-based models are therefore unlikely to have been caused by paracellular or facilitated transport occurring in the cell-based model. The most likely scenario is that the solubilized concentrations give a false indication of permeability that depends on the formulation used. Instead, the interactions of fenofibrate with the formulations is likely the important factor for mass transfer. At this point however, we cannot exclude that the formulations to some extent interact with the membranes. Both these factors – fenofibrate interaction with colloidal structures formed by the formulations and formulations potentially interacting with the membrane – are nonetheless highly likely to be influenced by the digestion of the formulations. They are therefore dependent on the digestive agent used, a parameter that is different between the LiDo and Caco-2 based lipolysis-permeation assays compared in this work.

In this instance, we have little reason to believe that the observed differences in mass transfer between models are the result of the membranes used. If the flux across the LiDo-based model membrane were significantly lower than that of Caco-2 for instance, we would have reason to assume that supersaturation kinetics could be a cause behind the observed differences. In our study, the flux was higher over the artificial membrane than over the cell-based. As discussed by Stillhart et al. (18), the absorption of lipolysis products can affect the supersaturation ratio of the drug if they are absorbed faster than the drug. A possible explanation could therefore exist if lipolysis products are absorbed to a much greater degree in the cell-based assay or *in vivo*. This would proportionally lower the solubilizing capacity of the type IIIA LBFs and make them more similar to the type IV.

## Conclusion

In this study, artificial membranes were studied to evaluate their use in predicting the performance of LBFs in lipolysis-permeation assays. Hexadecane membranes were found unsuitable for use with triglyceride-containing LBFs. Lecithin-based membranes (LiDo) with PVDF filter support were compatible with the formulations and the commonly used porcine pancreatin. The results of the study are inconclusive regarding the suitability of LiDo in the lipolysis-permeation assay. The model drug and formulations assayed, with porcine pancreatin as digestive agent, produced the same results as the state-of-the-art *in vitro* lipolysis assay. Further studies are required to characterize physical changes of membrane structure during digestion of LBFs, and investigate the cause of mass-transfer differences between the LiDo-based and the Caco-2 cell-based method. Due to the minimal incubation time required in this method, we expect that it could also be suitable for cell-free head-to-head comparisons of other supersaturating formulations, such as amorphous solid dispersions.

## Electronic supplementary material

ESM 1 (PDF 758 kb)

## Abbreviations

API: Active Pharmaceutical Ingredient
AUC: Area Under the Curve
BCS: Biopharmaceutics Classification System
BSA: Bovine Serum Albumin
DS-PAMPA: Double-Sink Parallel Artificial Membrane Permeability Assay
FaSSIF: Fasted State Simulated Intestinal Fluid
HDM: Hexadecane Membrane
IVIVR: *In Vitro In Vivo* Relationship
LBF: Lipid-Based Formulation
LC: Long-Chain
LiDo: Lecithin-in-Dodecane
LY: Lucifer Yellow
MC: Medium-Chain
NME: New Molecular Entity
SDS: Sodium Dodecyl Sulfate
PB: Phosphate Buffer
PBS: Phosphate Buffered Saline
PVDF: Polyvinylidene Difluoride
