# Supplementary material for: Suitability of Artificial Membranes in Lipolysis-Permeation Assays of Oral Lipid-Based Formulations
Source: Pharm Res. 2020 May 20;37(6):99. doi: 10.1007/s11095-020-02833-9 (PMC7239831; doi:10.1007/s11095-020-02833-9)
Supplement: Supplementary file 1 — (PDF 758 kb) [file 11095_2020_2833_MOESM1_ESM.pdf]

Electronic Supplementary Material

Publication title:
Suitability of artificial membranes in lipolysis-permeation assays of oral lipid-based formulations

Authors:
Oliver J. Hedge[1], Christel A. S. Bergström[1,2*]

[1]Department of Pharmacy, Uppsala University, Sweden.
[2]The Swedish Drug Delivery Center, Department of Pharmacy, Uppsala University, Sweden.

*Corresponding author:
Christel Bergström
Department of Pharmacy
Husargatan 3, Box 453, SE-75123 Uppsala, Sweden.
Tel: +46 18-471 41 18 | Fax: +46 18-471 42 23
E-mail: christel.bergstrom@farmaci.uu.se

Table of contents:
ESM Table I 2
Fig. S1 2
Fig. S2 3
ESM Table II 3
Fig. S3 4
Fig. S4 5
Fig. S5 6
ESM References 7

Table S1 Lipophilic drug compounds approved as NMEs by the FDA between 2015–2019 (3), and comparison to historical datasets. Physicochemical properties predicted from structure (isomeric SMILES) using ADMET Predictor 9.0 (Simulations Plus, CA, USA).

**Year**	**S+logP > 5**		**MlogP > 4.15**		**S+logd$_{6.5}$ > 5**	
2015 – 2016	3	(12%)	1	(4%)	1	(4%)
2017	4	(19%)	0	(0%)	1	(5%)
2018	2	(7%)	4	(14%)	2	(7%)
2019	5	(24%)	1	(1%)	2	(10%)
2015 – 2019	14	(15%)	6	(8%)	6	(6%)
Pre-1990[a]	2	(3%)	2	(3%)	1	(2%)
USAN library[b]	N/A	-	225[c]	(10%)	N/A	-

[a]Database of 60 compounds with FDA approval prior to 1990 (4). [b]Database of 2245 compounds in Phase II clinical trials during the 1990s. [c]ClogP > 5 or MlogP > 4.15 (4). Abbreviations: unionized partition coefficient between octanol and water (logP); Moriguchi logP (MlogP); pH-dependent distribution coefficient between octanol and water at pH 6.5 (logD$_{6.5}$).

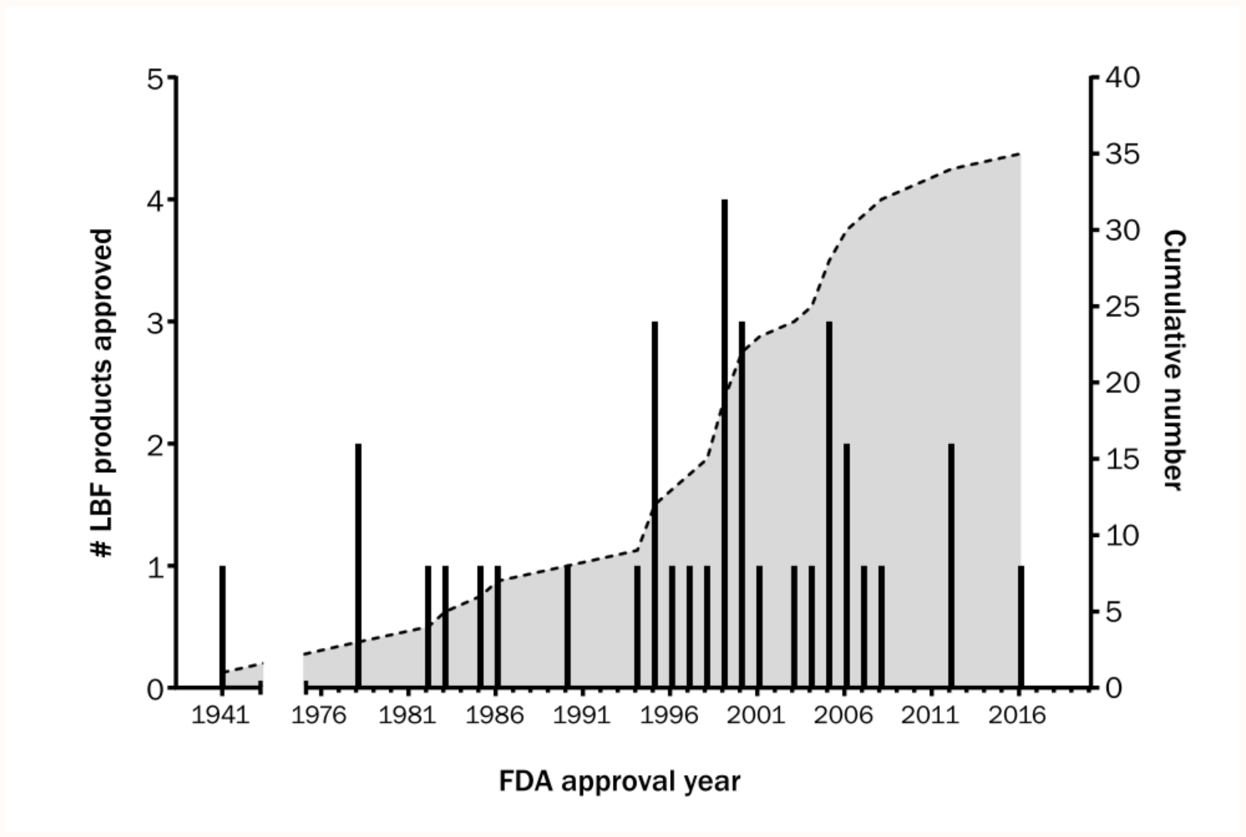

Fig. S1 Lipid based formulation (LBF) products approved by the FDA by year. Bars (to be read against the left y-axis) indicate the number of LBFs approved that year, and the dotted line (to be read against the right y-axis) indicates the cumulative number of approved products.

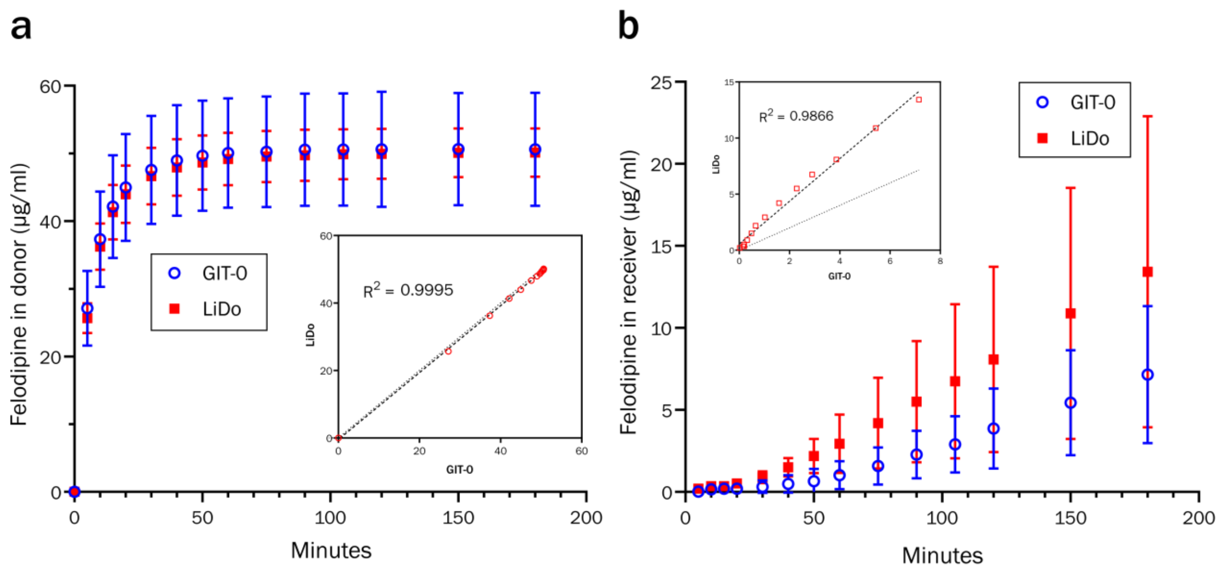

Fig. S2 (**a**) Dissolution of felodipine in FaSSIF and (**b)** permeation measured *in situ* by UV probes in the µFlux system (Pion Inc.). Blue circles are GIT-0 lipid solution membranes (Pion Inc.) separating the compartments, while red squares are LiDo membranes (lecithin in *n*-dodecane, in-house). The inserts show dependent variables of parent graphs plotted against each other (red symbols), with linear regression in black and unity (y = x) is represented by the dotted gray lines.

Table S2 pH change during lipolysis at 37 °C of a type IIIB-MC lipid-based formulation dispersed in buffers of increasing Tris-maleate concentrations.

**Tris-maleate (mM)**	**pH at t=0**	**Min. pH**	**average pH (over 52 min)**	**ΔpH/mmol NaOH**
10	6.503	5.433	5.498	1.09
20	6.018	5.540	5.593	0.90
200	6.516	6.237	6.265	N/D

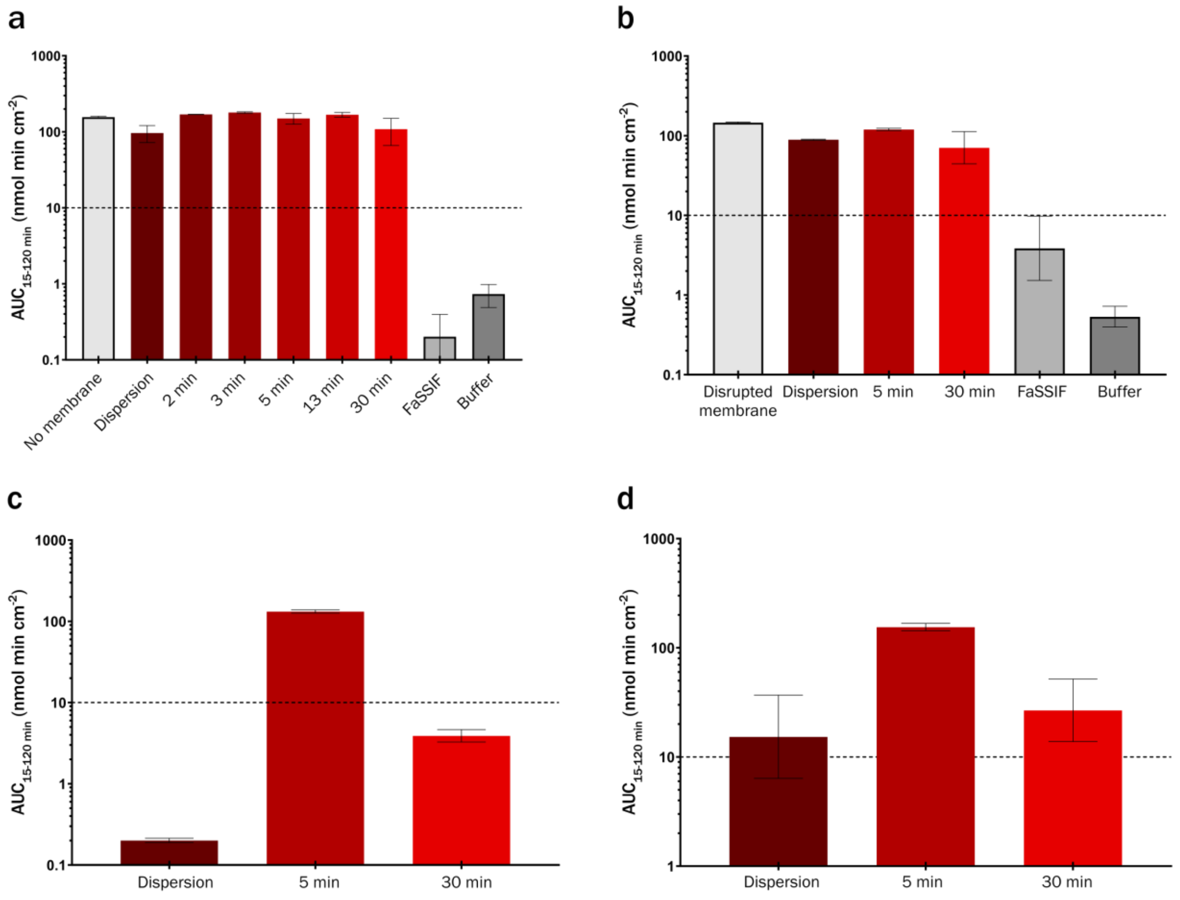

Fig. S3 Mass transfer of permeation marker Lucifer Yellow (AUC values, 15–120 min) through artificial membranes or naked polycarbonate filters (positive control) subjected to varying stages of LBF digestion (inhibited enzymes), FaSSIF or lipolysis buffer (negative controls). Six receiver samples were taken over two hours of membrane exposure to lipolysis media. (**a**) Hexadecane membranes (HDM), (**b**) GIT-0 membranes (soy lecithin in *n*-dodecane) with phosphate buffer (PB, 10 mM) in the receiver compartment, (**c**) GIT-0 membranes with Acceptor Sink Buffer (20 mM HEPES and 1% w/v SDS) in receiver compartment, and (**d**) GIT-0 membranes with PB supplemented with 4% (w/w) bovine serum albumin (BSA) in receiver compartment. The different shades are for visual clarity only.

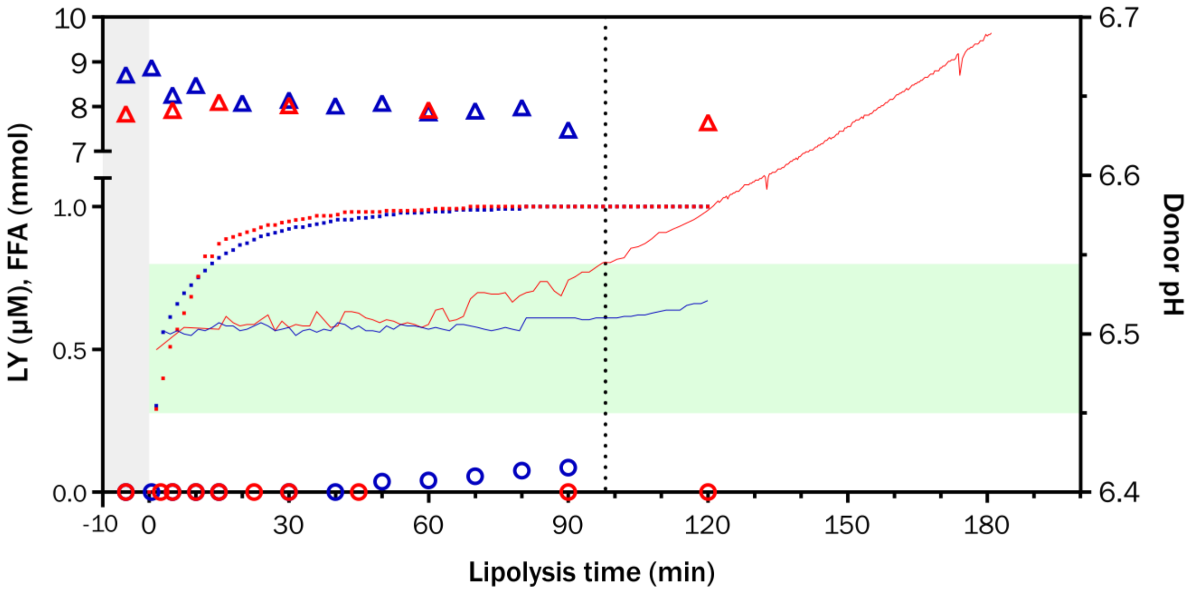

Fig. S4 Lucifer Yellow (LY) in the ENA system with GIT-0/PVDF membrane in two separate experiments (red and blue symbols) as a function of lipolysis time (x-axis). Donor compartment pH (right y-axis) was controlled with autotitration of 0.6 M NaOH solution into donor solution for pH < 6.5. The grey shaded area shows the dispersion time (10 min), before addition of porcine pancreatin (t = 0 min), during which no titration of NaOH occurred. Triangles show LY concentrations (µM) in the donor compartment and circles show LY concentration in receiver compartment. Donor solution pH is shown by the solid lines, in one experiment (red) exceeding acceptable parameters (green shaded area) after 98 min of digestion (vertical dotted line). The dotted curves represent the digestion of a type IIIB medium chain (C8-10) LBF as free fatty acid (FFA) release measured by NaOH titration (mmol). FFA values have been normalized to $n_{norm} = n_i - (n_{max} - 1)$, where n_{max} values corresponded to 0.7925 and 1.15 mmol for red and blue experiments, respectively.

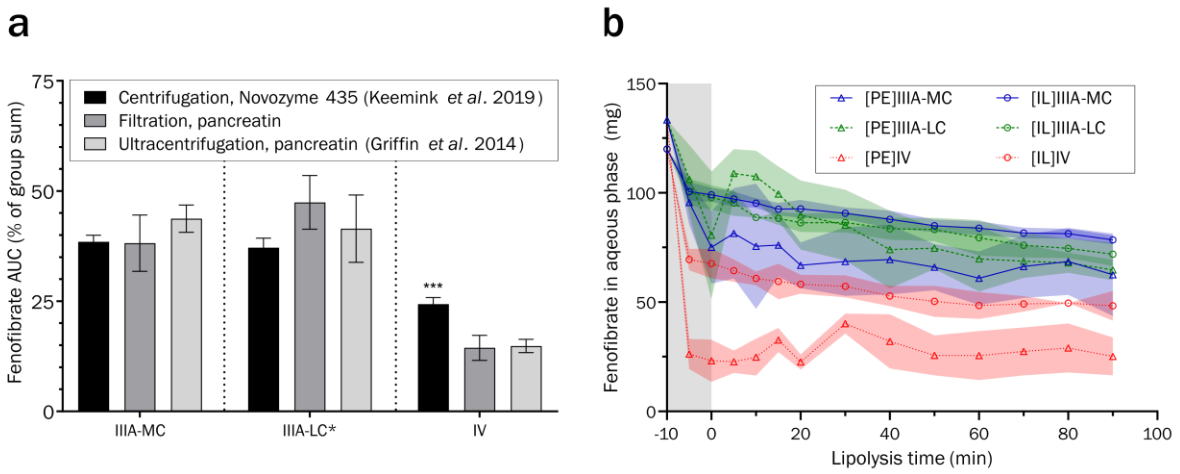

Fig. S5 Solubilized fractions of fenofibrate in different lipid-based formulations, comparisons with data from Keemink *et al.* 2019 and Griffin *et al.* 2014 (1,2). (**a**) Results presented as AUC of aqueous concentration curves over 60 min of digestion. AUC values have been normalized to percentage of group sum for comparability. Filtration was performed using 0.1 µm nylon syringe filters (Whatman PuraDisc 13). *Some samples from the initial 20 minutes of digestion were impossible to filter due to high backpressure, and were separated by centrifugation as per (1). (**b**) Results presented as mass of fenofibrate recovered from aqueous fractions (concentrations multiplied by total volume in donor vessel). Triangles indicate digestion by porcine pancreatin extract (PE, this work) and circles indicate digestion by Novozyme 435 (IL, Keemink *et al.*). Data points at t = -10 are the expected amounts, and the dispersion phase before addition of enzymes is indicated by the grey shaded region. Blue solid lines are the type IIIA-MC formulation, green dashed lines are type IIIA-LC, and red dotted lines are type IV. Colored shaded areas show the standard deviations. Abbreviations: medium chain (MC); long chain (LC).

ESM References

1. Keemink J, Mårtensson E, Bergström CAS. A lipolysis-permeation setup for simultaneous study of digestion and absorption in vitro. Mol Pharm. 2019 Jan 10;16(3):921–30.

2. Griffin BT, Kuentz M, Vertzoni M, Kostewicz ES, Fei Y, Faisal W, et al. Comparison of in vitro tests at various levels of complexity for the prediction of in vivo performance of lipid-based formulations: Case studies with fenofibrate. Eur J Pharm Biopharm. 2014 Apr;86(3):427–37.

3. U.S. Food and Drug Administration. New Drugs at FDA: CDER's New Molecular Entities and New Therapeutic Biological Products [Internet]. [cited 2019 Dec 16]. Available from: www.fda.gov/Drugs/DevelopmentApprovalProcess/DrugInnovation/

4. Lipinski CA, Lombardo F, Dominy BW, Feeney PJ. Experimental and computational approaches to estimate solubility and permeability in drug discovery and development settings. Adv Drug Deliv Rev. 1997 Jan;23(1–3):3–25.
